# Performance of Xpert HIV-1 viral load test in Senegal: a country of high circulation of CRF02_AG

**DOI:** 10.11604/pamj.2022.42.136.32041

**Published:** 2022-06-20

**Authors:** Pauline Yacine Sène, Halimatou Diop-Ndiaye, Aissatou Sow Ndoye, Brianan Kiernan, Khadidiatou Coulibaly, Ousseynou Ndiaye, Sada Diallo, Anna Julienne Selbe Ndiaye, Mengue Fall, Adjiratou Aissatou Ba, Makhtar Camara, Cheikh Saad Bouh Boye, Ndeye Fatou Ngom, Charlotte Lejeune, Cheikh Tidiane Ndour, Coumba Toure Kane

**Affiliations:** 1 Laboratoire de Bactériologie-Virologie CHU le Dantec, Université Cheikh Anta Diop de Dakar, Dakar, Sénégal,; 2 Clinton Health Access Initiative, Dakar, Senegal,; 3 Division de Lutte contre le SIDA/IST, Dakar, Sénégal,; 4 Institut de Recherche en Santé, de Surveillance Epidémiologique et de Formation, Dakar, Sénégal,; 5 United Nations International Children's Emergency Fund (UNICEF), Dakar, Sénégal

**Keywords:** HIV-1, plasma, viral load, point of care system, Senegal, CRF02_AG

## Abstract

**Introduction:**

the introduction of the point-of-care in HIV-1 viral load quantification appears to be a complementary strategy to the existing conventional system of the acceleration plan for the achievement of the three 90s in Senegal. The objective of this study was to evaluate the performance of the Xpert® HIV-1 viral load in the context of circulation of non-B, non-C subtypes.

**Methods:**

two hundred samples, were tested on Xpert® HIV-1 Viral Load using 1 ml of plasma in comparison to 600 μl on Abbott Real-time HIV-1 assay. The difference between viral load values was considered significant for Dlog <0.5 log copies/ml.

**Results:**

a good correlation (r=0.985) was noted and confirmed using passing-bablok regression (slope 1.048; 95% CI: 1.036 to 1.069) for 188 samples with samples. A mean difference of 0.0075 log10 copies/ml for a 95% confidence interval (CI) of 0.002 log10 copies/ml to 0.013 log10 copies/ml was obtained. Sensitivity and specificity were respectively 93.6% and 93.5% at the threshold of 1.6 log10 copies/ml and 100% and 99% at the threshold of 3.0 log10 copies/ml.

**Conclusion:**

these results show that Xpert® HIV-1 Viral Load has excellent performance. In Senegal, and can be used for HIV viral load monitoring.

## Introduction

Eliminating HIV/AIDS infection by 2030 requires the achievement of the intermediate United Nations Programme on HIV and AIDS (UNAIDS) 2020 targets of “90-90-90” and now UNAIDS 2025 targets of “95-95-95” which were to screen 90% and 95% of HIV-positive people, but 90% and 95% of HIV-positive people on antiretroviral treatment (ART) and ensure that 90% and 95% of people on ART had a suppressed viral load (VL) by 2020 and 2025, respectively [1,2]. The 3^rd^ “90” is a challenge in West and Central African countries, which evaluates ART effectiveness and virological failure identification at an early stage among patients [3]. In the region, viral load access is centralized, with the availability of viral load platforms only in national or regional laboratories. Access to viral load testing is difficult for populations in remote areas, due to sample transport challenges that lead to delays in results return to health facilities and to patients. These long delays in processing samples and returning results often result in a high number of patients lost to follow-up [4]. In Senegal, the monitoring of VL measurement still remains a major challenge. In 2017, out of a total number of 42,915 people living with HIV (PLHIV) on ART, only 27% had benefited from a viral load measurement and 19% of those who received a viral load test were virologically suppressed (CV < 1000 copies/ml) [5]. Point-of-Care (POC) technologies such as GeneXpert offer an innovative approach to viral load surveillance, allowing real access to molecular biology in decentralized structures, especially in countries with limited resources [6]. This technology is widely used in the molecular diagnosis of tuberculosis in decentralized laboratories [7].

The integration of the HIV-1 VL test with the Xpert® HIV-1 viral load (Cepheid) to the GeneXpert platform has improved the return of results on the same day, and better monitoring of ART, adherence counseling, and rapid ART line change services when ART resistance is suspected [8,9]. For VL determination, several techniques, such as the Xpert® HIV-1 viral load, are available and have been developed from subtype B [10], which represents about 10% of HIV strains worldwide. Subtype B is found mainly in Europe and northern countries, while non-B subtypes predominate worldwide. In Africa, many studies have evaluated the Xpert® HIV-1 viral load, especially in Eastern and Southern African countries [11,12], where the molecular epidemiology of HIV-1 strains is homogeneous with mainly the circulation of subtype A, D [13], and C [14,15]. However, studies have shown that some variants are missed or under-quantified by some VL tests such as CRF02_AG, which is predominant in Senegal and other West African countries [16-18]. Despite its low HIV prevalence, Senegal is characterized by high genetic diversity, with a circulation of many variants of HIV. CRF02 AG accounts for approximately 60% of HIV strains in the general population and sex workers [19]. This great diversity of HIV-1 strains is known to have a real impact on the performance of techniques, hence the need to carry out validation before the introduction of a new technique in the context of variant circulation [20,21]. Therefore, the objective of this study was to compare the performance of the Xpert® HIV-1 Viral Load test for the quantification of the plasma viral load of HIV-1 in Senegal, a context of high genetic diversity and Abbott Real-time HIV-1® (Abbott molecular diagnostics, Wiesbaden, Germany), defined as reference technique.

## Methods

**Sampling:** the evaluation included a panel of 100 plasma samples from the biobank of the Laboratory of Bacteriology-Virology of the Aristide le Dantec Hospital, from which 40% had been chosen on the basis of a VL value greater than 3 log copies/ml measured by Abbott Real-time HIV-1 test. This was supplemented by an additional 100 whole blood samples collected in EDTA tubes from HIV-1 infected and consenting patients as part of their follow-up visit. All patients were consented and over 18 years old and had been receiving treatment for at least 06 months. The study does not include patients with serious medical conditions. The plasmas were obtained by centrifugation of the blood tubes at 2,500 rpm for 10 min, then stored at -80°C until the tests were carried out.

**HIV-1 viral load quantitation:** the viral quantitation was performed for each platform according to the manufacturer´s instructions. Abbott Real-time HIV-1 assay performed on m2000sp/rt is an in vitro qRT- PCR targeting the integrated region of the highly conserved pol gene. This assay detects HIV-1 group M, N, O, and P and several Chronic renal failure (CRF). A fully automatic extraction was performed with the Abbott m2000sp, and the amplification coupled with real-time detection was realized with the Abbott m2000rt. The assay dynamic range is 1.60-7.0 log10 copies/ml for 600 µl of plasma samples [22]. The Xpert® HIV-1 Viral Load was also performed according to the manufacturer's instructions on the 04-module platform of the GeneXpert (Cepheid) machine with 1 ml of plasma as input volume and a linear detection range from 1.60 to 7.0 log copies/ml [23].

**Genotyping and phylogenetic analysis:** discordant samples with a VL ≥ 3.0 log were genotyped in the pol gene. The entire protease gene and a fragment, encoding the 240 first amino acid of the RT gene, were amplified by a nested RT-PCR using the HIV French drug resistance technique [24] PCR products were purified (Invitrogen- Thermo Fisher scientific) and directly sequenced on Seq Studio™ Genetic analyzer system. The generated sequences were edited using the recall (beta v3.05)- web-based sequence analysis. HIV-1 subtype and CRF designations were determined by phylogenetic analysis. The nucleotide sequences were aligned using a neighbor-joining method with 100 bootstrap replicates, as implemented in the Sea view software [25]. All pure subtypes and CRFs available in the Los Alamos database and circulating in West Africa were included in this analysis, and some CRF02-AG of Senegal from Gen Bank were added as references and aligned.

**Statistical analysis:** differences between VL were considered as significant when the value was greater than 0.5 log/copies/ml. For the comparison of VL platform quantitation, correlation and concordance tests were determined between Abbott Real-time HIV-1 assay and Xpert® HIV-1 viral load with MethVal software (method validator software 1.1.9.0, Philippe Marquis, Metz, France). Data obtained by the Xpert® HIV-1 viral load were compared to the Abbott Real-time HIV-1 assay data by linear regression analysis and coefficient of variation. Bland-Altman statistical bias method was used to determine the level of agreement between the results obtained by the new and the standard systems. Ninety-five percent confidence intervals were used for the analysis. For qualitative values measurement between two assays, at 1.60 log copies/ml and 3 log copies/ml, sensitivity and specificity were calculated. We used the Cohen´s kappa coefficient, a statistic that measures inter-rater agreement for qualitative values between two assays. The kappa coefficient indicates a satisfying agreement between 0.6 and 0.8, and an excellent agreement above 0.8 [26].

**Ethical and regulatory considerations:** the study protocol was approved by the National Study Committee for Health Research of Senegal (Reference: Protocol SEN 18/48) and the administrative authorization was issued by the Senegalese Ministry of Health and Social Action.

## Results

**Figure 1:**
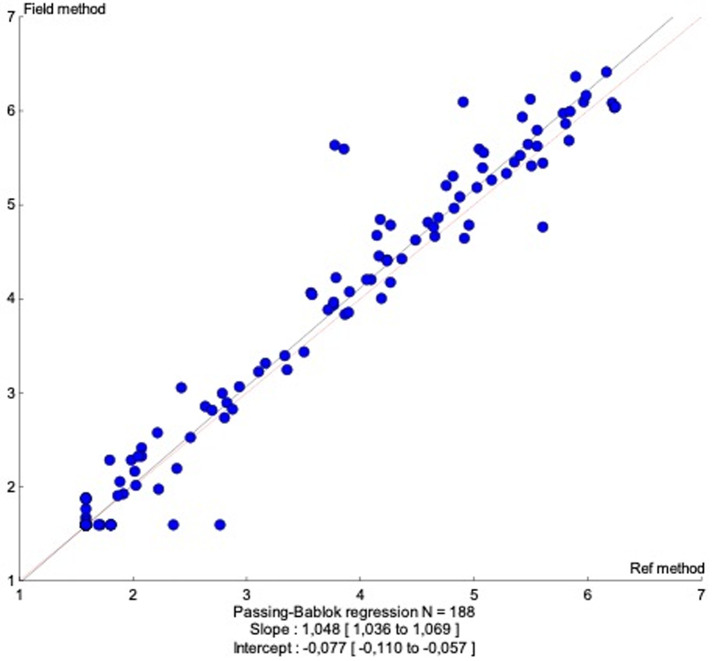
passing-Bablok regression for Xpert® HIV-1 viral load and the Abbott Real-time HIV-1 assay

**Figure 2:**
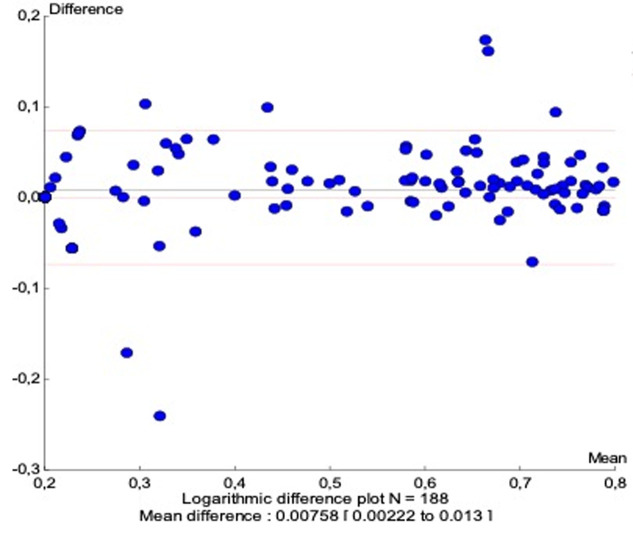
bland-Altman plot comparing Xpert® HIV-1 viral load against the Abbott Real-time HIV-1 assay

**Figure 3:**
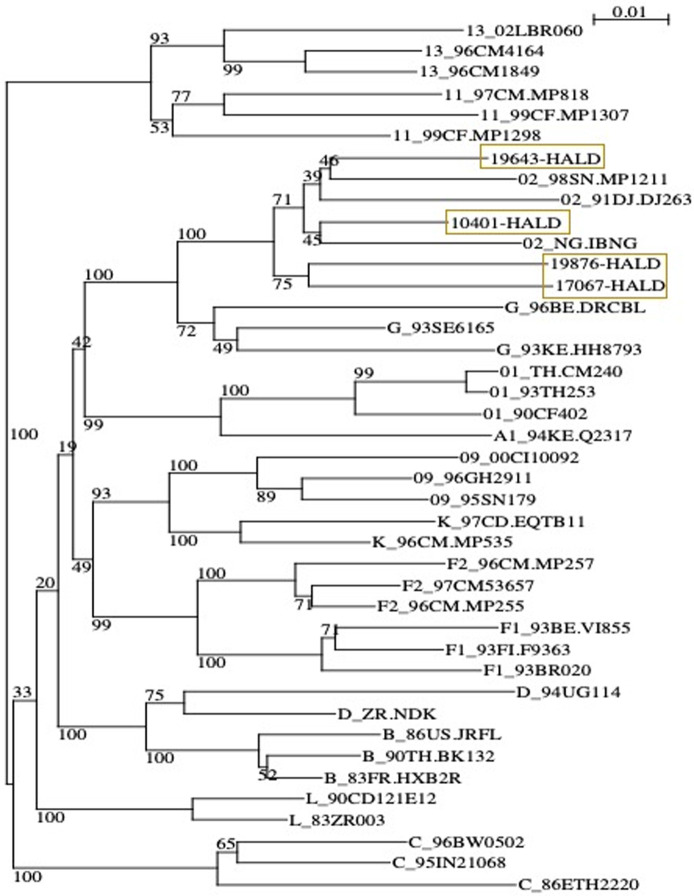
phylogenetic tree of samples with D-log< 0.5 log copies/ml

To compare VL results between Abbott Real-time HIV-1 assay and Xpert® HIV-1 viral load, 200 samples were tested on both platforms and results were obtained for 188 samples while 12 samples were invalid (6%). A significant correlation was observed between the HIV-1 RNA levels obtained by the two assays, with r=0.985 (Figure 1). This correlation was confirmed using the Passing-Bablok regression (intercept -0.077; 95% confidence interval [CI], -0.110 to -0.057) and a slope of 1.048 (95% CI, 1.036 to 1.069) (Figure 1) were noted. Similarities between the two assays were evaluated by the Bland-Altman plot method (Figure 1). The overall mean difference in the HIV-1 RNA values obtained by Xpert® HIV-1 Viral Load and Abbott assay was 0.007 (95% confidence interval [CI], 0.002 to 0.013) (Figure 2). Using a threshold of 40 copies/ml (1.6 log10 copies/ml) for detectable HIV-1 RNA load, the assay's agreement was 86% with a sensitivity of 93.6% (Table 1). The means of VL were 2.798 log10 copies/ml for Abbott Real-time HIV-1 assay and 2.876 log10 copies/ml for Xpert® HIV-1 Viral Load, and 12 samples presented a Dlog > 0.5 log copies/ml. Out of these 12 samples, 09 were overestimated by the Xpert® HIV-1 Viral Load and 01 was overestimated by the Abbott Real-time HIV-1 test; two detectable samples with a low viral load value by the Abbott Real-time HIV-1 test were undetectable with the Xpert® HIV-1 Viral Load. (Table 2). Using a threshold of 1,000 copies/ml (3.0 log10 copies/ml) for clinical monitoring of ART, the assays were in agreement at 97% with a sensitivity of 100%. (Table 1). Discordant samples with Dlog > 0.5 log were sequenced in pol gene and revealed 04 CRF02_AG strains (Figure 3).

**Table 1:** agreement on detection of HIV-1 RNA between Xpert® HIV-1 Viral Load and Abbott real-time HIV-1 assay at two detection levels, 1.6 log10 copies/ml and 3.0 log10 copies/ml

	Abbott assay detectability by HIV-1 RNA level			
Detection threshold for Xpert HIV-1 VL assay	1.6 log10 copies/ml		3 log10 copies/ml	
	**Undetectable**	**Detectable**	**Undetectable**	**Detectable**
Undetectable	87	6	121	0
Detectable	6	89	1	66
Total	93	95	122	66

* Using a threshold of 40 copies/ml (1.6 log10 copies/ml) for detectable HIV-1 RNA load, the assays agreement was 86% with a sensitivity of 93.6% *Using a threshold of 1,000 copies/ml (3.0 log10 copies/ ml) for clinical monitoring of ART, the assays were in agreement at 97% with a sensitivity of 100%

**Table 2:** discordant samples with D-log >0.5 log copies/ml

Id patient	Log Vl Abbott	Log Vl Xpert	D-log
17067-HALD*	3.78	5.63	-1.84
12300-HALD*	3.86	5.59	-1.73
21897-HALD*	4.91	6.09	-1.18
19852-HALD *	4.18	4.84	-0.67
21875-HALD *	5.5	6.12	-0.62
21963-HALD*	2.43	3.05	-0.62
19876-HALD*	5.05	5.59	-0.54
10401-HALD*	4.15	4.67	-0.52
21877-HALD *	4.27	4.78	-0.51
18425-HALD**	2.36	1.59	0.77
19643-HALD**	5.61	4.76	0.85
L-122**	2.77	1.59	1.18

* Overestimated by Xpert® HIV-1 viral load; **Overestimated by Abbott rea- time HIV-1

## Discussion

The use of Point of care (POC) testing can improve the clinical management of PLHIV by significantly reducing the turnaround time for healthcare workers to receive patient results and take clinical action [27-29]. Therefore, POC and near-POC are recommended by new WHO clinical guidelines for viral load monitoring and early detection of virological failure [30]. The objective of this study was to evaluate the performance of Xpert® HIV-1 Viral Load for the achievement of the 3^rd^ “90” in Senegal, a country with a high diversity of HIV-1. Indeed, many studies in Senegal highlighted the great diversity of HIV-1 strains with a predominance of non-B and non-C subtypes, mainly circulating recombinant forms. CRF02_AG remains the predominant strain in the country with more than 55% over the years [19,31,32]. This evaluation, carried out in this context, showed however an excellent correlation and concordance between platforms with a Pearson coefficient r=0.985 and a mean difference of 0.0075 log10 copies/ml. These results are in line with studies performed in South African countries where subtype C is predominant [33,34]. In addition, Xpert® HIV-1 Viral Load offers also comparable performance to the Abbott Real-time HIV-1 test at clinical values of interest. An excellent agreement (Kappa: 86%) was found at 1.6 log copies/ml (detectability threshold) as well as at 3 log copies/ml (Kappa: 97%), the virological failure threshold [35], according to WHO recommendations [30].

Despite this good correlation between the two tests, some samples presented significant differences with D-log greater than 0.5 log10 cp/ml with an overestimation of viral load values by the Xpert® HIV-1 Viral Load. This could be due to the lower input volume used in Abbott test comparing to Xpert® HIV-1 Viral Load as the ratio was 600 versus 1000µl. However, several studies highlighted Abbott HIV-1 Real-time viral load underestimation at the lower level of viremia (under 3 log copies/ml) [36,37]. To better understand these discrepancies, sequencing was carried out and, showed CRF02_AG strains despite the low number of samples analyzed (4 CRF02_AG out of 4 samples sequenced). This could be a limit of this study, but a larger sampling study comparing Xpert® HIV-1 viral load and Cobas TaqMan have underlined the failure of Xpert® HIV-1 viral load to detect some CRF02_AG strains from Nigeria with high viral load titers [38]. Nevertheless, the good performance of the Xpert® HIV-1 viral load requires also adequate training to decrease the rate of errors. In our study, 6% of the samples were invalid and required another test. These errors occurred primarily at the beginning of the study and may be attributable to user errors as described by other authors [11]. However, the rate of error was not higher than in other studies even if invalid tests were not tested again due to the limited number of cartridges available for this study. Our study confirmed the usefulness of the Xpert® HIV-1 Viral Load tests for the quantification of VL in Senegal. Therefore, the use of the Xpert® HIV-1 Viral Load will improve same-day VL and result return, which could allow immediate assessment of virological failure. Improving access to this test will support the prioritization of patients for adherence counseling, reduce loss to follow-up and improve early detection of virological failure to avoid ART line switching [39]. The achievement of the UNAIDS 90-90-90 targets depends on HIV surveillance and preventing a future epidemic of ART-resistant HIV strains that could delay these goals in sub-Saharan Africa [40].

## Conclusion

Due to the simplicity, rapid results, and good performance of the Xpert® HIV-1 viral load, the test can support decentralizing VL surveillance from specialized laboratories to local hospitals and clinics as part of their routine clinical care. This will help achieve the ambitious goals to achieve the third 90, which becomes 95 for 2025, across the region.
